# Differences in Kinematic Changes From Self-Selected to Fast Speed Gait in Asymptomatic Adults With Radiological Signs of Femoro-Acetabular Impingement

**DOI:** 10.7759/cureus.43733

**Published:** 2023-08-18

**Authors:** Fares Yared, Abir Massaad, Ziad Bakouny, Joeffroy Otayek, Aren-Joe Bizdikian, Joe Ghanimeh, Chris Labaki, Diane Ghanem, Ismat Ghanem, Wafa Skalli, Ayman Assi

**Affiliations:** 1 Laboratory of Biomechanics and Medical Imaging, Saint Joseph University of Beirut, Beirut, LBN; 2 Institut de Biomécanique Humaine Georges Charpak, Arts et Métiers ParisTech, Paris, FRA

**Keywords:** walking speed, kinematics, 3d reconstruction, gait, hip, femoro-acetabular impingement

## Abstract

Femoro-acetabular impingement (FAI) may present as alterations in the skeletal morphology of the hip. Repercussions of FAI can be witnessed in self-selected speed walking as well as physical exercise such as running or fast speed walking. The aim of this study was to investigate changes in kinematics at different gait speeds in subjects presenting with radiological findings invoking FAI. One hundred thirty asymptomatic adults underwent biplanar X-rays with a calculation of 3D hip parameters: acetabular anteversion, abduction and tilt, vertical center edge angle (VCE), femoral anteversion, neck-shaft angle, acetabular coverage of the femoral head, femoral head diameter and neck length. Parameters were classified according to FAI clinical thresholds. Two groups were created: Control group (63 subjects having up to one subnormal hip parameter in favour of FAI) and Radiographic FAI group (67 subjects having ≥2 subnormal hip parameters that might cause FAI). All subjects underwent 3D gait analysis at self-selected and fast speed, from which kinematic parameters were generated. Arithmetic differences between fast and self-selected speed gait were considered as gait changes. Subjects in the Radiographic FAI group had decreased acetabular tilt (24 vs. 19˚), anteversion (19 vs. 16˚), abduction (55 vs. 53˚), femoral anteversion (18 vs. 14˚) and increased VCE (29 vs. 33˚, all p<0.05), compared to controls. Changes from self-selected to fast speed showed that subjects in the Radiographic FAI group had lower range of motion (ROM) pelvic rotation (7 vs. 4˚) and ROM hip flexion/extension (10 vs. 7˚), reduced hip extension (-4 vs. -2˚) and step length (16 vs. 13 cm; all p<0.05). The Radiographic FAI group had decreased acetabular abduction, anteversion and femoral anteversion in favour of FAI. When adapting from self-selected to fast speed gait, the Radiographic FAI group seemed to limit pelvic rotation and hip flexion/extension resulting in a decrease in step length. These kinematic limitations were previously reported in subjects with symptomatic FAI. Gait analysis could be considered as a functional diagnostic tool to assess FAI along with radiological assessment.

## Introduction

Femoro-acetabular impingement (FAI) may present as alterations in the skeletal morphology of the hip [1]. These modifications are widely considered to be one etiology of hip osteoarthritis. FAI may present as multiple types: CAM, pincer, and combined. The CAM may present as a small femoral head, reduced femoral neck offset, or as a bony bump over the superolateral aspect of the femoral neck [2]. The occurrence of symptoms in the case of CAM is mostly seen in men with a mean age of 32 years, whereas pincer FAI is usually seen in symptomatic women with a mean age of 40 years [1]. Moreover, FAI has also been reported in athletes: the hypothesized etiology is that athletes tend to push their bodies over the physiological limit by excessive exercising [3]. Overall, in athletic and non-athletic subjects, FAI is diagnosed through a combination of clinical and radiological findings [1].

Radiographs of patients with FAI often show an increased alpha angle, increased acetabular depth, and increased lateral center edge angle, as well as the crossover sign indicating focal acetabular retroversion [4]. The alpha angle is measured on a Dunn view radiograph between the femoral neck axis and a line connecting the center of the femoral head with the point where the head-neck contour sphericity begins. The acetabular depth is defined as the relative position of the ilio-ischial line to both the medial wall of the acetabulum and the femoral head. The lateral center edge angle is measured between a vertical line and a line connecting the femoral head center with the lateral edge of the acetabulum. A crossover sign is found when the anterior acetabular wall overlaps the posterior wall.

Three-dimensional radiography through CT-scan or MRI has been shown to be important in diagnosing FAI [5].

MRI is a relevant imaging technique to assess chondrolabral damage. In addition, 3D CT scans being a low-cost technique are usually recommended for diagnosis and surgical planning in patients with FAI [6]. However, these modalities present with their own limitations, such as high doses of radiation in the case of CT scans, and are both acquired in the supine position. This will alter the position and morphology of the hip compared to the standing position, which is the functional situation of most of daily living activities. Recently, low-dose biplanar X-rays have shown their advantages in accurately assessing 3D hip morphology in the standing position [7-9]. Furthermore, biplanar X-ray assessment in a loaded position could be beneficial for later arthroscopic investigations of the hip.

The usual clinical presentation of FAI patients is chronic hip pain and reduced range of motion. Interestingly, previous studies have shown that impingement occurs in both symptomatic and asymptomatic subjects [10, 11], with pincer FAI being the most common subtype among asymptomatic subjects. In addition, asymptomatic subjects showed radiological signs of FAI regardless of whether pain was present.

Repercussions of FAI can be witnessed in self-selected speed walking as well as physical exercise such as running or fast speed walking [12]. Three-dimensional gait analysis was used in the context of FAI in the kinematic assessment of both symptomatic and previously diagnosed FAI patients [13] and post-arthroscopic follow-up of operated FAI patients [14]. A previously reported kinematic comparison between symptomatic FAI and control subjects reported scarce and unclear differences when walking at a normal speed [15]; thus, kinematic deficits could be better assessed in further functional tasks that challenge hip kinetics, such as walking at a fast speed gait.

The research question was: are there any differences in gait kinematics between asymptomatic subjects presenting radiological FAI hip signs and those who do not, when walking at self-selected and fast speed?

Therefore, the aim of this study was to investigate changes in kinematics at different gait speeds in subjects presenting radiological hip signs that might cause FAI.

## Materials and methods

Study design

This is a cross-sectional IRB (Ethical Committee, Saint Joseph University: #CEHDF285) approved study. All methods were carried out in accordance with relevant guidelines and regulations. Asymptomatic subjects were recruited from the students, staff and faculty members of our institution, according to the following inclusion criteria: no previous history of orthopedic surgery, no scoliosis or leg length discrepancy, no hip pain or prior history of femoro-acetabular impingement. All subjects signed a written informed consent form. Informed consent from all subjects for the publication of identifying images was obtained.

Data acquisition

A total of 130 asymptomatic subjects (65 females and 65 males) were included. The mean age was 29.5±11 years ranging from 18 to 60 years. For each subject, demographic characteristics were anonymously collected and stored in a data system: age, gender, weight and height, using a digital weight scale and a stadiometer.

Three-dimensional gait analysis

**Figure 1:**
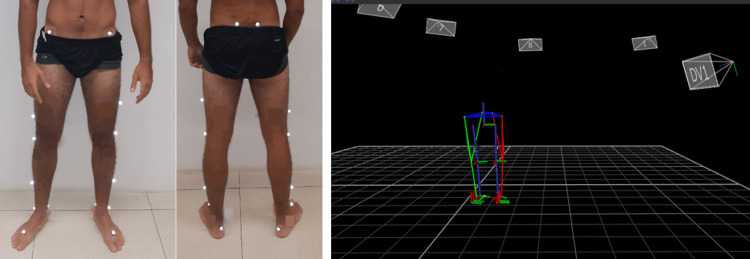
Placement of the reflecting markers based on the modified Helen Hayes protocol. The Plug-in Gait model was applied to calculate the lower limb kinematics

Each subject underwent three-dimensional gait analysis (3DGA) using a VICON (VICON Motion Systems, Oxford, UK) optoelectronic motion system (7 MX3+ infrared cameras, with 200 Hz frequency). Marker placement was based on the modified Helen Hayes protocol and the Plug-in Gait model was applied (Figure 1) [16]. Subjects were asked to walk at a self-selected speed along a 10-m walkway. Then, they were asked to walk at a fast speed gait along the walkway. In this second part of the acquisition, the participants were asked to walk at a maximal walking speed without running. Data was processed using the pipeline in Workstation (VICON Motion Systems, Oxford, UK): fill gap routine 10 frames and Woltring filter with a scale of 10.

Several walking trials were recorded for each participant and the kinematic curves for all trials were overlaid within a single graph using Polygon software (VICON Motion Systems, Oxford, UK). Based on a visual observation, the most repeatable trial was identified and subsequently one gait cycle associated to this trial was selected. This gait cycle is considered representative of the subject’s walking profile and used for the analysis [17, 18].

One representative trial for each type of gait was then used to calculate three-dimensional joint angles and time-distance parameters. Kinematic parameters of the pelvis, hip, knee and ankle were then calculated in MATLAB (MathWorks, Natick, USA) for each subject in the frontal, sagittal and horizontal planes [19, 20]. Maximum, minimum, mean and range of motion (ROM) were calculated on each kinematic waveform describing the joint or segment angle during the gait cycle. The following time-distance parameters were also calculated: walking speed (m/s), cadence (steps/min), foot off (% of gait cycle), single support(s) and step length (m). Single support was normalized to stride time and was expressed as a percentage of the whole gait cycle. All kinematic and time-distance parameters generated in this study have been previously defined in the literature [20, 21].

Gait alterations

Gait changes, related to kinematics and time-distance parameters, between self-selected and fast speed gait were determined by calculating the arithmetic difference between both conditions for each subject and gait parameter (value of gait parameters during fast speed gait minus value of gait parameters in self-selected speed).

Biplanar radiography

All subjects underwent a full body biplanar X-ray exam (EOS Imaging, Paris, France) right after the 3DGA acquisition, which was performed by a senior radiology technician and under the supervision of the researchers. Subjects were asked to stand upright in a standardized position [22, 23].

**Figure 2:**
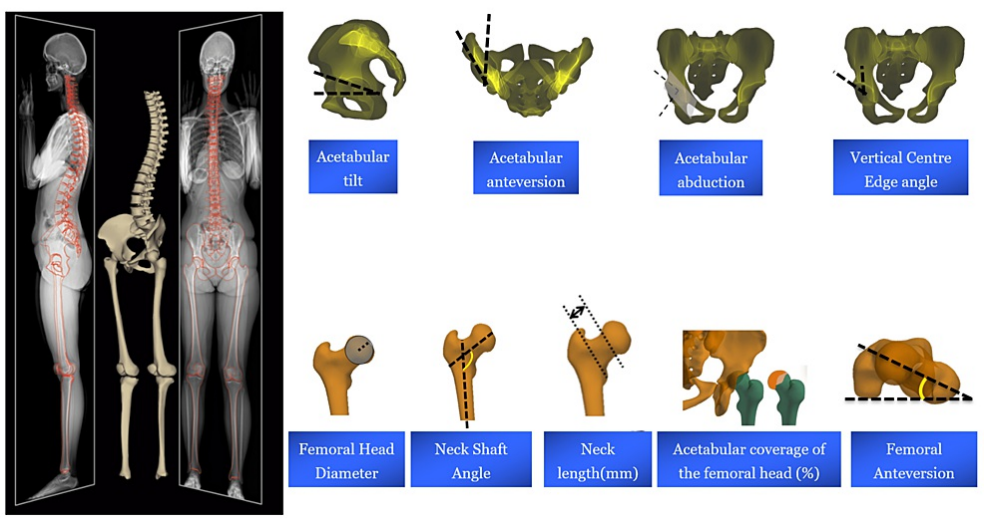
Hip radiological parameters based on 3D reconstructions from biplanar X-rays

Subjects’ pelvises and proximal femurs were reconstructed in 3D using a specific software (Arts et Métiers ParisTech, Paris, France). The following 3D radiological hip parameters (Figure 2) were generated: acetabular tilt (°), acetabular anteversion (°), acetabular abduction (°), 3D vertical center edge angle (VCE; °), femoral anteversion (°), neck-shaft angle (NSA) (°), acetabular coverage of the femoral head (%), femoral head diameter (mm) and neck length (mm). Validity and reliability of the aforementioned 3D hip radiological parameters were previously evaluated [8, 24, 25].

Based on the established standards reported in the literature, the following hip parameters were considered to be within the assumed normal range if: acetabular anteversion >15° [26]; VCE <34° [27]; Neck-shaft angle >135° [28] and femoral anteversion >15° [28]. As for the other hip parameters, since no normative data exist in the literature, the assumed normal range was considered to be the mean±1SD.

The values of the nine hip radiological parameters were assessed for each subject. If a value was found to be outside of the assumed normal range, the 3D radiological hip parameter was considered to be subnormal, and thus underlying asymptomatic femoro-acetabular impingement (FAI). Values of acetabular tilt, acetabular abduction, femoral head diameter and neck length were considered subnormal if they were inferior to the established interval of [Mean-1SD], since their decrease can be associated with FAI, based on hip geometry [29-31]. Values of acetabular coverage of the femoral head were considered subnormal if they were superior to the established interval of [Mean+1SD]. The quantitative assessment of these parameters was performed in order to subdivide the sample into two groups. Asymptomatic subjects having up to one subnormal 3D radiological parameter formed the Control group. The Radiographic FAI group consisted of asymptomatic subjects having at least two subnormal hip parameters and considered as presenting hip signs that might be associated with FAI.

Statistical analysis

Statistical analysis was conducted separately for the left and right side. Since the results were comparable, the left side was considered for the analysis. Data were compared between the Control group (the non-FAI) and the Radiographic FAI group. Sex was compared between the two groups using Fisher’s exact test. As for the other demographic factors (height, weight and age) as well as the 3D radiological hip parameters, comparisons were computed using Student’s t-test after verification of normality (Shapiro-Wilk’s test). Gait kinematics of self-selected speed, fast speed and gait changes were compared between both groups using ANCOVA, followed by Bonferroni correction, while controlling for the potentially confounding demographic factors (Age). For time-distance parameters, height was controlled while comparing between both groups. Statistically significant differences were discussed only when the difference exceeded the level of uncertainty of the considered parameter, 5° for the radiological parameters and 2 to 3° for the kinematic parameters [21, 32-34]. A post hoc power analysis was performed using G*Power (v.3.1.9.4). Statistical analysis was performed using XLSTAT version 2020.1.3 (Addinsoft, Paris, France). The level of significance was set at 0.05.

A qualitative description between cases, one from each group, was conducted in order to observe differences in 3D hip morphology and kinematic waveforms.

## Results

All demographic parameters are displayed in Table 1. After grading the hip radiological parameters for each asymptomatic subject, 63 subjects had up to one subnormal parameter and were therefore included in the Control group. The remaining 67 subjects had more than two subnormal parameters and were therefore included in the Radiographic FAI group. The distribution was as follows: subjects with no subnormal parameters (N=18), with 1, 2, 3, 4 and 5 subnormal parameters (N=45, N=37, N=17, N=8 and N=5, respectively). Only age showed a significant difference (p<0.04) among demographic factors: the Radiographic FAI group subjects had a mean age of 27±10 years, lower than the Control group subjects who had a mean age of 32±12 years.

**Table 1:** Demographic parameters of the whole sample, Control group and Radiographic FAI group *bold: statistically significant result

Demographic parameter	Total (N=130)	Control group (N=63)	Radiographic FAI group (N=67)	Comparison (p-value)
Age (years)	29.5±11	32±12	27±10	0.04*
Height (cm)	169.6±10	170±10	168±11	0.2
Weight (kg)	71±15	73±14	69±15	0.056
Sex	65 F/65 M	28 F/35 M	37 F/30 M	0.22

Comparisons of 3D radiological hip parameters

**Figure 3:**
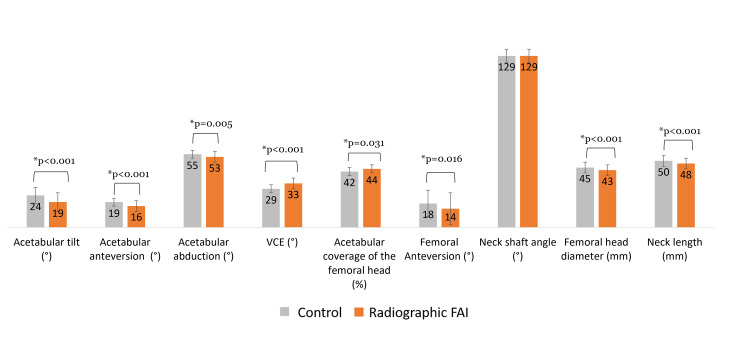
Comparison of hip parameters between the Control group and the Radiographic FAI group. Error bars refer to standard deviation.

Comparisons of 3D radiological hip parameters (Figure 3) between the two groups showed that subjects in the Radiographic FAI group had a significant decreased acetabular tilt (Control group: 24±6° vs. Radiographic FAI group: 19±7°; p<0.001), anteversion (Control group: 19±4° vs. Radiographic FAI group: 16±4°; p<0.001), abduction (Control group: 55±3° vs. Radiographic FAI group: 53±4°; p=0.005), femoral anteversion (Control group: 18±10° vs. Radiographic FAI group: 14±12°; p=0.016), femoral head diameter (Control group: 45±4 mm vs. Radiographic FAI group: 43±4 mm; p=0.011) and neck length (Control group: 51±4 vs. Radiographic FAI group: 48±5; p=0.006). Subjects in the Radiographic FAI group had a significantly increased VCE (Control group: 29±3° vs. Radiographic FAI group: 33±4°; p<0.001) and acetabular coverage of the femoral head (Control group: 42±3% vs. Radiographic FAI group: 44±3%; p=0.031).

Gait kinematics and time-distance parameters

Gait kinematics and time-distance parameter changes were similar between both groups at self-selected speed. During fast speed gait, only ROM pelvic rotation changes were different between both groups (p=0.017): The Control group had higher ROM pelvic rotation changes (20±6°) compared to that of the Radiographic FAI group (17±6°).

**Figure 4:**
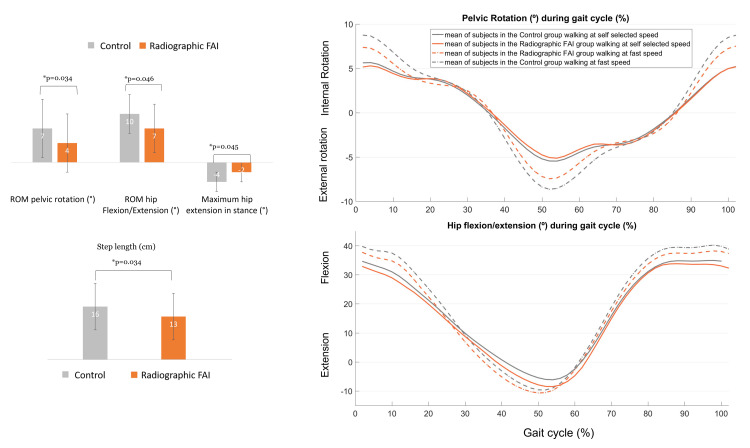
Comparison of gait changes from self-selected to fast speed between the Control group and the Radiographic FAI group. Error bars refer to standard deviation.

During gait changes from self-selected to fast-speed gait, significantly different kinematic and time-distance parameters were found between both groups (Figure 4). The Radiographic FAI group had lower ROM pelvic rotation changes (Control group: 7±6° vs. Radiographic FAI group: 4±6°; p=0.034), decreased hip extension in stance changes (Control group: -4±3° vs. Radiographic FAI group: -2±3°; p=0.045) and shorter step length changes (Control group: 16±0.07 cm vs. Radiographic FAI group: 13±0.07 cm; p=0.034). Comparisons of kinematics of the pelvis and hip and time-distance parameters between both groups at self-selected and fast speed gait as well as the changes between speeds are displayed in Table 2.

**Table 2:** Comparison of gait kinematics and time-distance parameters between the Control group and the Radiographic FAI group at different walking speeds. *bold: statistically significant result that exceeds the level of uncertainty

Kinematic and time-distance parameters	Self-selected speed			Fast speed			Gait alteration (Δ fast–self-selected speed)		
	Control group	Radiographic FAI group	p-value	Control group	Radiographic FAI group	p-value	Control group	Radiographic FAI group	p-value
ROM pelvic tilt (°)	3.2±1	3.3±1	0.10	4.7±1	4.3±1	0.15	1.4±1	1±1	0.20
ROM pelvic obliquity (°)	9.5±4	10.8±4	0.06	13.9±5	13.8±5	0.06	4±3	3±3	0.01
ROM pelvic rotation (°)	12.8±5	12.3±5	0.80	20±6	17±6	0.01*	7±6	4±6	0.034*
Mean pelvic tilt (°)	12±5	11.4±6	0.005	13.5±6	13.1±6	0.004	1.6±2	1.6±2	0.79
Mean pelvic obliquity (°)	0.3±1	0.1±1	0.07	0.3±2	-0.1±2	0.10	0±1	0±1	0.38
Mean pelvic rotation (°)	0±3	0.6±2	<0.001	0±3	0.5±2	<0.001	0±2	0±2	0.38
ROM hip flexion/extension (°)	42±4	43±5	0.14	51.4±5	51±6	0.30	10±6	7±5	0.086
ROM hip abduction/adduction (°)	13.2±3	14.5±4	<0.001	17.7±5	18.2±4	0.002	4.5±3	3.7±3	0.21
ROM hip internal/external rotation (°)	32.6±13	32±12	<0.001	34.3±14	33.3±13	<0.001	1.7±8	1.3±9	0.89
Mean hip flexion/ extension (°)	16.7±6	15.3±7	<0.001	18.7±7	17.5±7	<0.001	2±2	2±2	0.89
Mean hip abduction adduction	-1±3	-1±3	<0.001	-1.7±3	-1.37±3	<0.001	-0.7±1	-0.4±1	0.75
Mean hip internal/external rotation (°)	0.7±10	-1.5±7	0.36	-0.4±10	-2.1±8	0.55	-1.1±4	-0.6±2	0.59
Hip Extension in stance (°)	-7.1±7	-9.0±7	0.20	-11±8	-12±7	0.20	-4±3	-2±3	0.045*
Step Length (cm)	66±7	66±6	0.38	80±8	79±8	0.30	16±7	13±8	0.034*
Walking speed (m/s)	1.3±0.2	1.3±0.1	0.66	2±0.2	2±0.2	0.73	0.7±0.2	0.7±0.2	0.59
Cadence (steps/min)	114.5±10	116.8±8	0.038	147.4±12	151.8±14	0.016	32.8±11	35±10	0.39

**Figure 5:**
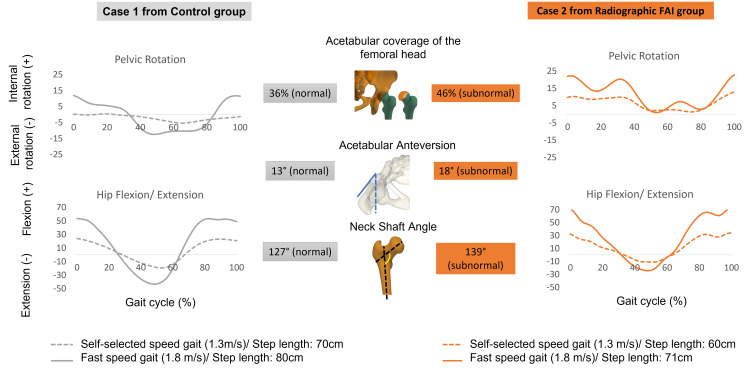
Example of internal/external pelvic rotation and hip flexion/extension changes from self-selected to fast speed gait in the Control group and the Radiographic FAI group

The qualitative comparison between one subject from the Control group (presenting with one subnormal parameter), and another subject from the Radiographic FAI group (presenting with three subnormal parameters) showed that these latter present an increased acetabular coverage, anteversion and NSA and a decreased changes in the ROM of the pelvic rotation, hip flexion/extension and a decreased step length during the fast-walking speed (Figure 5).

The calculated standardized effect of the radiological and kinematic parameters varied between 0.5 and 1.3 based on the signal and noise calculations [8, 21, 32-35]. The achieved power varied between 88 and 98%.

## Discussion

Femoro-acetabular impingement (FAI) is a prevalent condition commonly found in young patients [36]. This condition could potentially elucidate the later onset of hip osteoarthritis, a condition that was previously considered to be idiopathic. It has been shown to be a potential cause of acetabular cartilage and labral lesions. These morphologic alterations could subsequently lead to hip osteoarthritis [37]. Moreover, FAI has garnered even more interest since it has been described among asymptomatic subjects [10] as well as athletes [3, 10].

This study compared gait kinematics in self-selected, fast-speed gait and changes between self-selected and fast-speed gait between two groups of asymptomatic subjects divided based on 3D hip radiological parameters.

Radiological differences

Subjects classified in the Radiographic FAI group (displaying more than two subnormal 3D radiological hip parameters) had a significantly decreased acetabular anteversion, abduction, tilt and femoral anteversion. In addition, subjects in the Radiographic FAI group had a significantly increased VCE and acetabular coverage of the femoral head, thus increasing the risk of developing FAI [29, 30]. Moreover, the results in our study regarding femoral anteversion and acetabular coverage are in accordance with those reported in the literature in subjects with asymptomatic FAI: Audenaert et al. reported a decreased femoral anteversion and an overcoverage of the femoral head by the acetabulum which contribute to the risk of early collision between the proximal femur and the acetabulum. Thus, these findings could be predictive of clinical FAI [31].

In the Radiographic FAI group, the decreased acetabular abduction could, at least partly, explain the increased VCE and lateral coverage, which might explain the increase in the coverage of the femoral head by the acetabulum.

A study by Nehme et al. [38] reported that asymptomatic subjects presented with variations in the hip joint space width prior to the usual radiographic alterations. Having found significant differences in several 3D hip radiological parameters could address the importance of biplanar X-ray in establishing early subclinical changes in subjects from the general population having no pain related to FAI. This exam can be used as a screening tool in the athletic population.

Gait kinematics and time-distance parameters

No differences were found in self-selected speed between both groups. This lack of differences between the two groups could be explained by the fact that all the subjects in our study were asymptomatic, including those who had a hip radiographic sign that might cause FAI. The only significant difference in fast-speed gait was found when comparing the ROM pelvic rotation. The Radiographic FAI group showed a decreased ROM pelvic rotation. This could be explained by the acetabular overcoverage and the decreased acetabular anteversion. The combination of these two variations could predispose these subjects to FAI. A reduced pelvic axial rotation might be needed in order to avoid hitting the acetabular wall. This is in accordance with what has been previously reported in a systematic review regarding the pelvic compensatory mechanism in FAI subjects, mainly manifested to avoid pain or discomfort [13].

When adapting from self-selected to fast speed gait, the Radiographic FAI group had significantly decreased changes of the ROM pelvic rotation, maximum hip extension in stance and step length, as shown in the example in Figure 5. The ROM hip flexion/extension was reduced without reaching the level of significance. Even when controlling for the gait speed, the same results of kinematics were obtained. Compared to control subjects, the ROM of pelvic rotation, hip flexion and extension were significantly smaller in the Radiographic FAI group. In fact, gait adaptation alterations from self-selected to fast speed gait require an increase in ranges of motion at the levels of the pelvis, hip, knee and ankle in the three planes [39]. The increased VCE in the Radiographic FAI group leads to an increased lateral coverage which could explain the significant decrease of the ROM pelvic rotation. Furthermore, the overcoverage of the femoral head by the acetabulum in the Radiographic FAI group could possibly lead to a significant decrease in maximal hip extension, as a compensatory mechanism to avoid the hip impingement. The latter could consequently decrease significantly the ROM of hip flexion/extension and consequently lead to step length changes.

Although differences in gait between both groups were not high, these differences were statistically significant and higher than the uncertainty thresholds defined for each kinematic parameter [21, 40]. Moreover, it was expected that differences found in 3D hip radiological parameters and kinematic changes between both groups would not be high since all the subjects were asymptomatic.

Interestingly, differences found in 3D hip morphology and kinematic changes among the Radiographic FAI group were previously reported in symptomatic FAI patients. Hunt et al. found that patients with FAI exhibited significantly less maximum hip extension and adduction with associated changes in flexion and external rotation moments [41].

The similar kinematic results found in both asymptomatic subjects with hip radiographic morphology that might cause FAI [38] and symptomatic FAI subjects lead to the hypothesis that these changes in gait kinematics could precede the onset of symptoms of FAI patients. Furthermore, the decreased step length represents a possible consequence of the overall radiological changes which lead to a significant change in the gait profile of the Radiographic FAI group subjects.

Since it has been previously reported that FAI is one of the possible causes of hip osteoarthritis [36, 42, 43], gait analysis and 3D hip reconstruction based on biplanar X-rays could be possible predictors of early hip morphological and dynamic alterations. 3D gait analysis might serve as a predictor of FAI disease onset in previously asymptomatic subjects exhibiting subnormal 3D hip morphology. Therefore, an early detection of FAI signs based either on a 3D gait analysis or a radiographic exam, might be of great importance in sports professionals, especially for athletes like runners. This could potentially pave the way in the future for a preventive intervention related to coping movement strategies or even related to an orthopedic treatment.

This study presents several limitations. A symptomatic FAI group of patients was lacking in the current study. Future studies should integrate symptomatic subjects with FAI, presenting pain and altered radiographic parameters and asymptomatic subjects with no altered radiographic parameters in order to identify kinematic and 3D radiological discrepancies, that could then be used as predictors for symptomatic FAI.

The combination of normative 3D radiological hip parameters values with an interval of Mean±1SD in hip parameters whose normative values are not yet established could have served as a limitation; it is still not demonstrated that a specific 3D radiological parameter was shown to influence progression towards symptomatic FAI.

Future studies should include comparisons of efficacy in diagnostic assessment in FAI between biplanar X-rays and CT-scan. Additionally, longitudinal studies including patients presenting radiological and kinematic signs in favor of FAI, are needed to ascertain whether they will manifest FAI symptoms. Moreover, accounting for diverse confounding variables such as lifestyle, daily routine or sports engagement, will provide a more comprehensive understanding of this pathology. Furthermore, kinematic patterns should be compared between asymptomatic subjects having asymptomatic and subnormal 3D hip morphology with symptomatic and diagnosed FAI patients as well as asymptomatic subjects having no subnormal hip morphology.

## Conclusions

In conclusion, at self-selected speed, asymptomatic subjects within the Radiographic FAI group, presenting with an altered hip geometry, showed normal gait parameters. However, they were not able to show appropriate kinematic adaptations when they were asked to walk at a fast speed, which is comparable to symptomatic FAI subjects. Thus kinematic alterations might highly be expected in more complex tasks, or during sports activities, in athletes or non-athletes. Therefore, detecting FAI early signs in daily routine practice, by sports physicians, could lead to implement a preventive treatment in the future, particularly among sports professionals.
